# Supplementary figures and images for: MiR172-APETALA2-like genes integrate vernalization and plant age to control flowering time in wheat
Source: PLoS Genet. 2022 Apr 25;18(4):e1010157. doi: 10.1371/journal.pgen.1010157 (PMC9037917; doi:10.1371/journal.pgen.1010157)

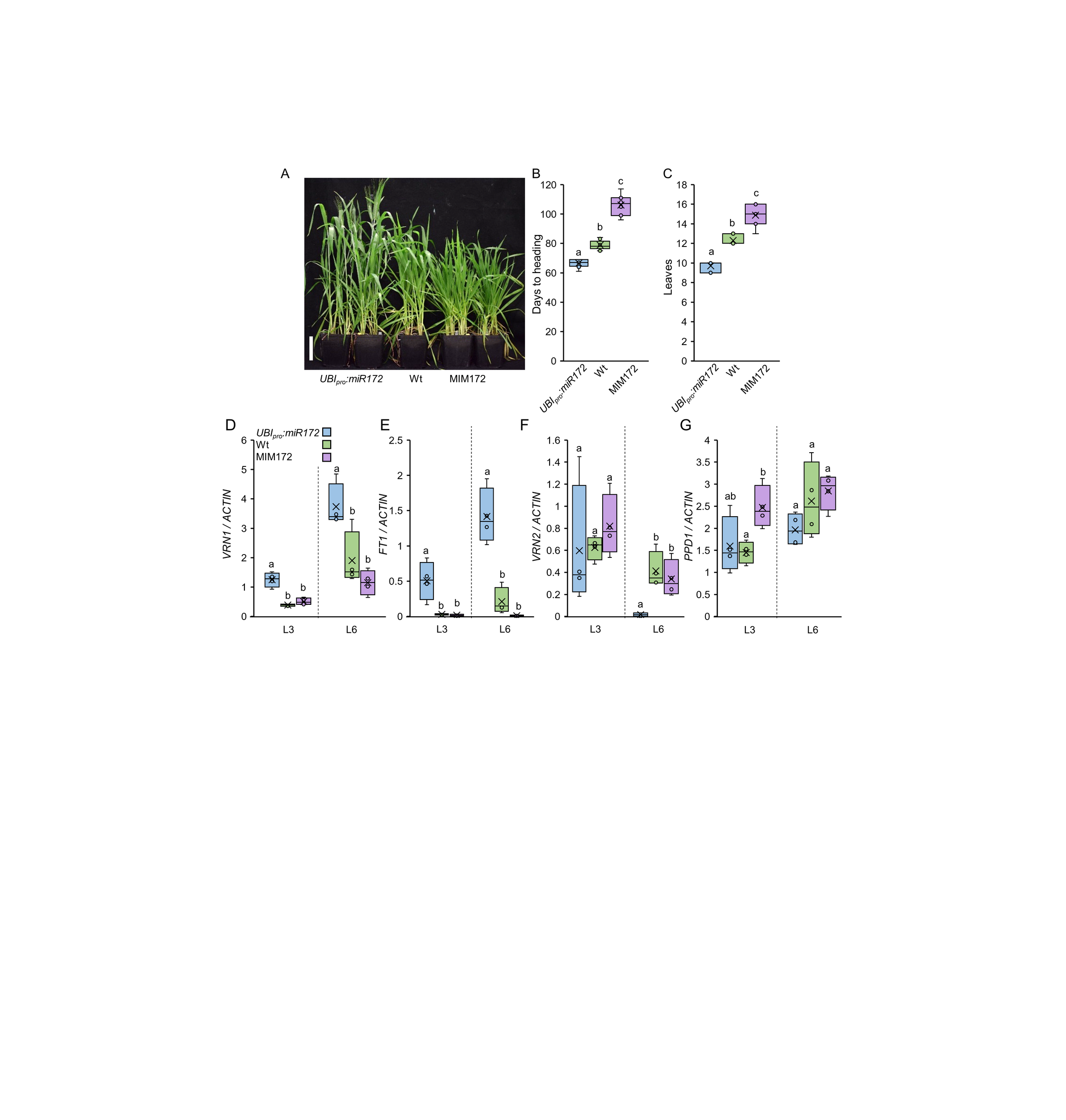

Supplement: S1 Fig — (A) Nine-week-old UBIpro:miR172, wild type Kronos (Wt) and MIM172 plants grown under SD. Scale bar = 10 cm. (B-C) Box plots showing days to heading (B; n ≥ 7) and the number of leaves produced by the main tiller (C; n ≥ 9) in UBIpro:miR172, wild type Kronos (Wt) and MIM172 plants grown under SD. (D-G) Box plots showing expression levels of VRN1 (D), FT1 (E), VRN2 (F), and PPD1 (G) determined by qRT-PCR in the 3rd (L3) and 6th (L6) leaves of UBIpro:miR172, wild type Kronos (Wt) and MIM172 plants growing under SD. ACTIN was used as an internal reference. Data correspond to four independent biological replicates. Different letters above the box plots indicate significant differences based on Tukey tests (P < 0.05). Raw data and statistical tests in Data H in S1 Data. (TIF) [file pgen.1010157.s004.tif]

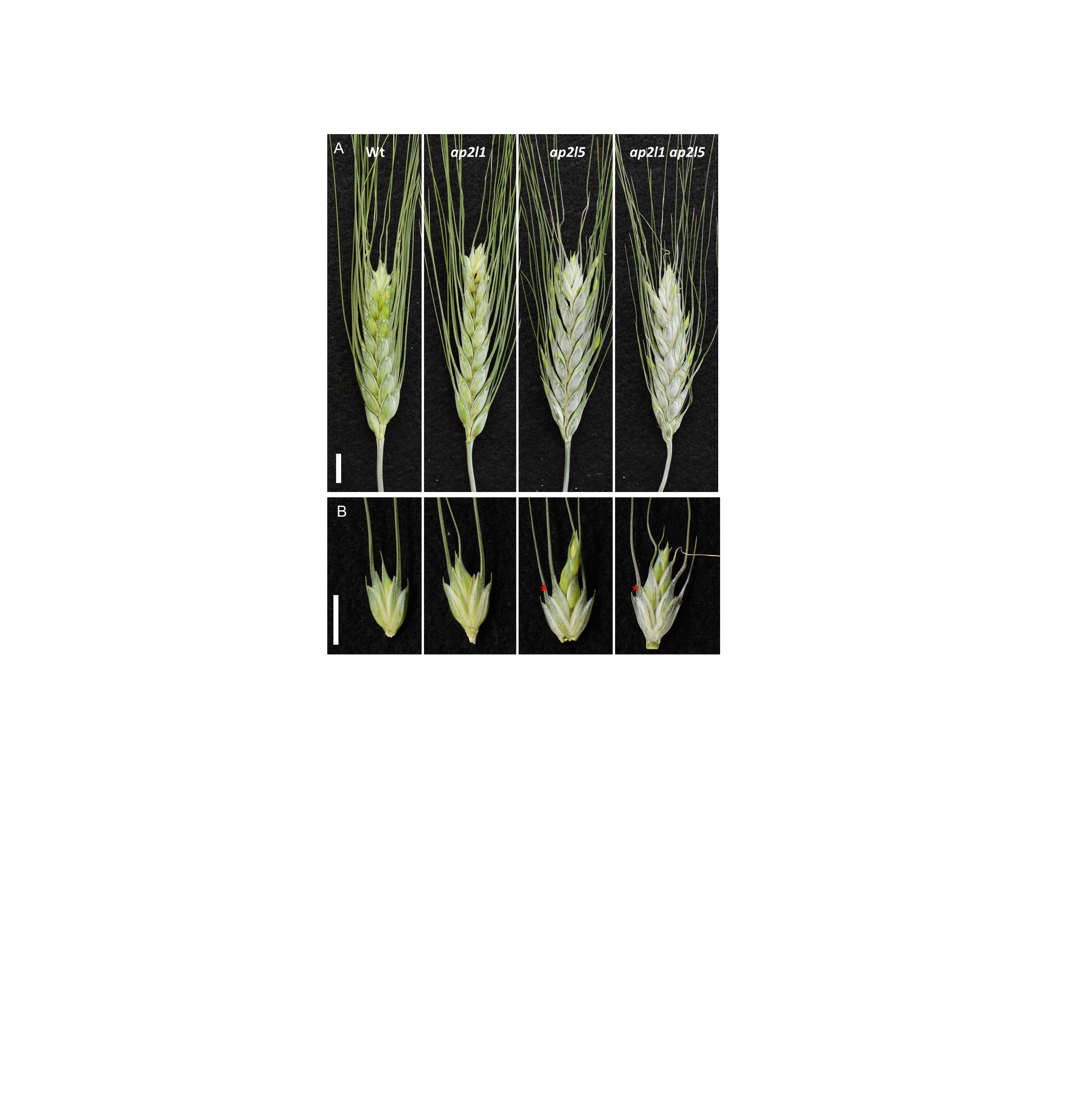

Supplement: S2 Fig — (A) Main spikes and (B) central spikelets from wild type Kronos (Wt), ap2l1, ap2l5 and ap2l1 ap2l5 plants. Red asterisks in ap2l5 and ap2l1 ap2l5 spikelets indicate first empty lemmas. (TIF) [file pgen.1010157.s005.tif]

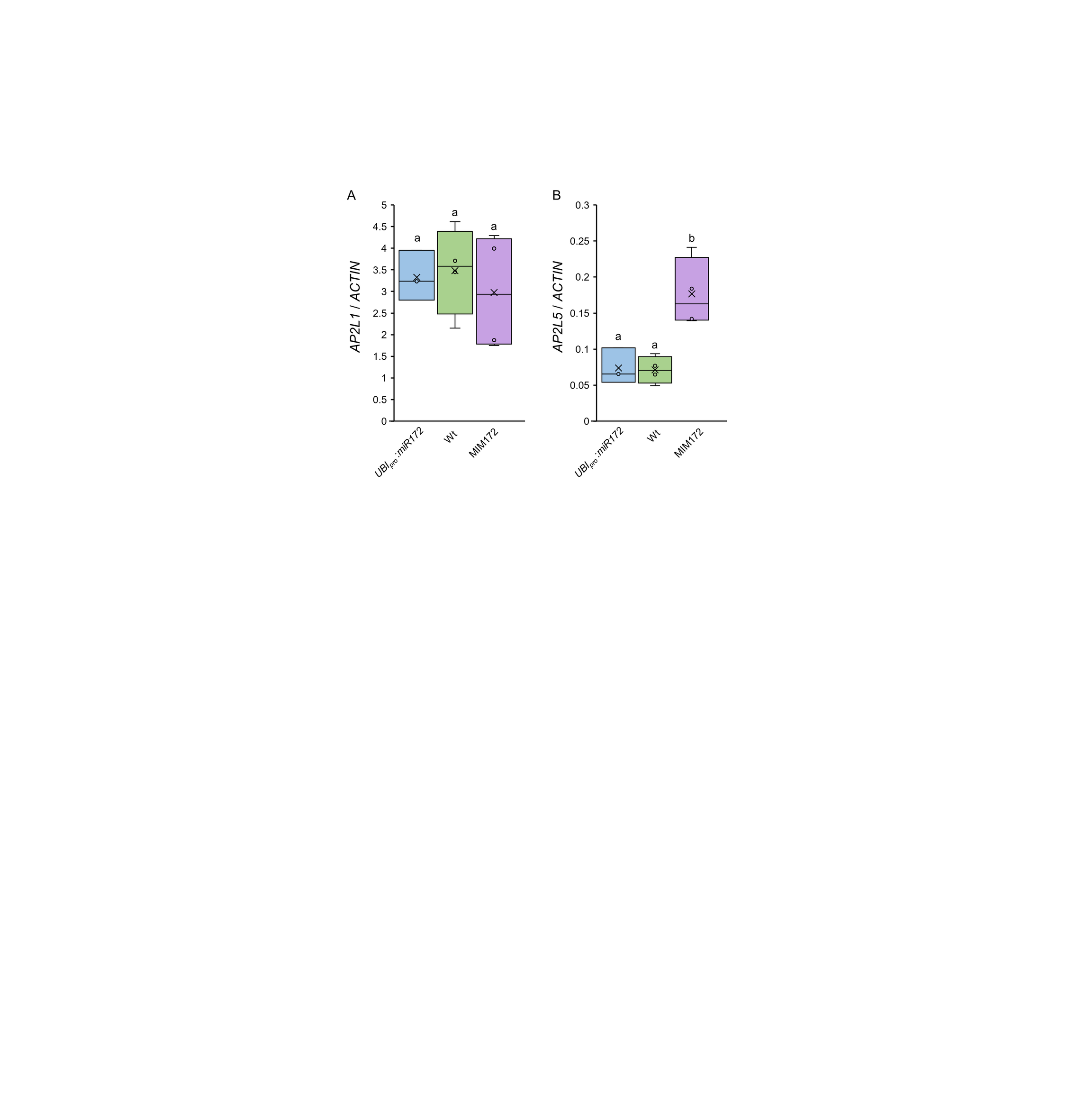

Supplement: S3 Fig — (A-B) Box plots showing AP2L1 (A) and AP2L5 (B) expression levels in the 5th leaf of UBIpro:miR172, wild type Kronos (Wt) and MIM172 plants grown under LD (same biological samples used to quantify miR172 expression in Fig 1B). Transcript levels were determined by qRT-PCR using ACTIN as internal reference. Data correspond to four independent biological replicates. Different letters above the box plots indicate significant differences based on Tukey tests (P < 0.05). Raw data and statistical tests in Data I in S1 Data. (TIF) [file pgen.1010157.s006.tif]

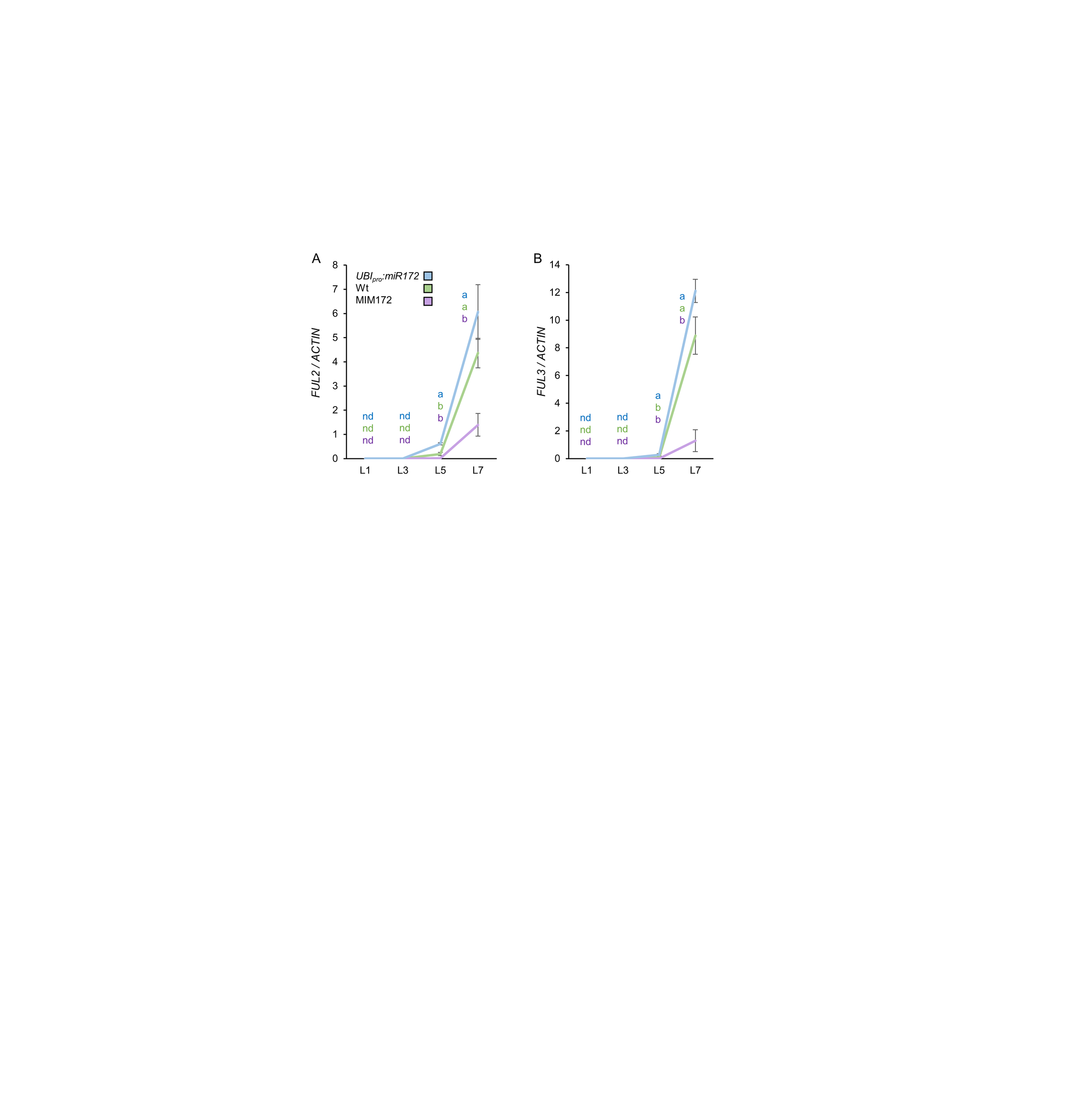

Supplement: S4 Fig — (A-B) Expression levels of FUL2 (A) and FUL3 (B) determined by qRT-PCR in the 1st (L1), 3rd (L3), 5th (L5) and 7th (L7) leaves of UBIpro:miR172, wild type Kronos (Wt) and MIM172 plants grown under LD. ACTIN was used as internal reference. Data correspond to four independent biological replicates. Different letters above the data point for each leaf indicate significant differences based on Tukey tests (P < 0.05). The color of the letter corresponds to the color of the genotype. Raw data and statistical tests in Data J in S1 Data. (TIF) [file pgen.1010157.s007.tif]

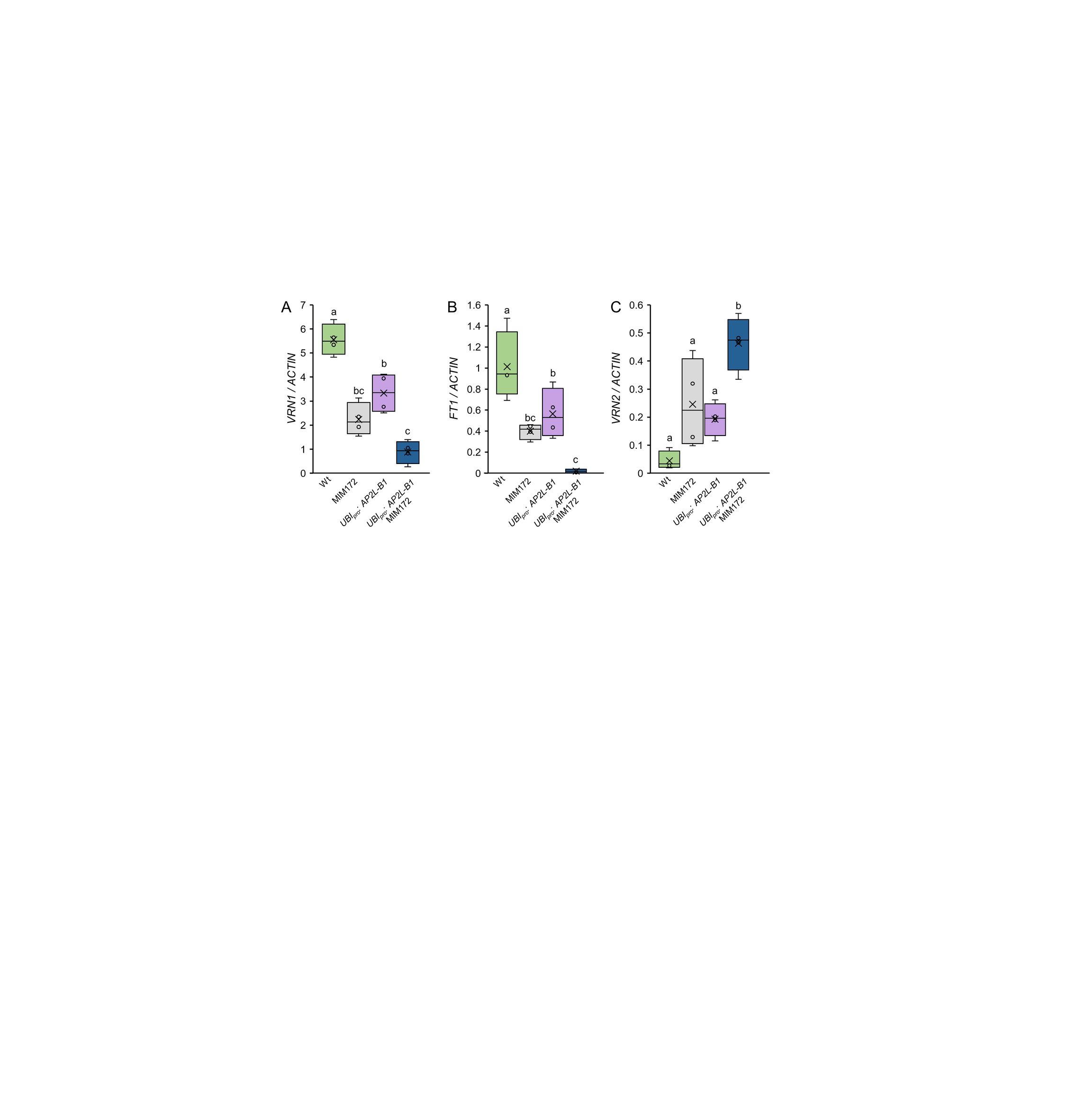

Supplement: S5 Fig — (A-C) Box plots showing the expression levels of VRN1 (A), FT1 (B) and VRN2 (C) determined by qRT-PCR in the 5th leaf of F2 plants segregating for UBIpro:AP2L-B1 and MIM172 transgenes grown under LD. ACTIN was used as internal reference. Data correspond to four independent biological replicates. Different letters above the box plots indicate significant differences based on Tukey tests (P < 0.05). Raw data and statistical tests in Data K in S1 Data. (TIF) [file pgen.1010157.s008.tif]

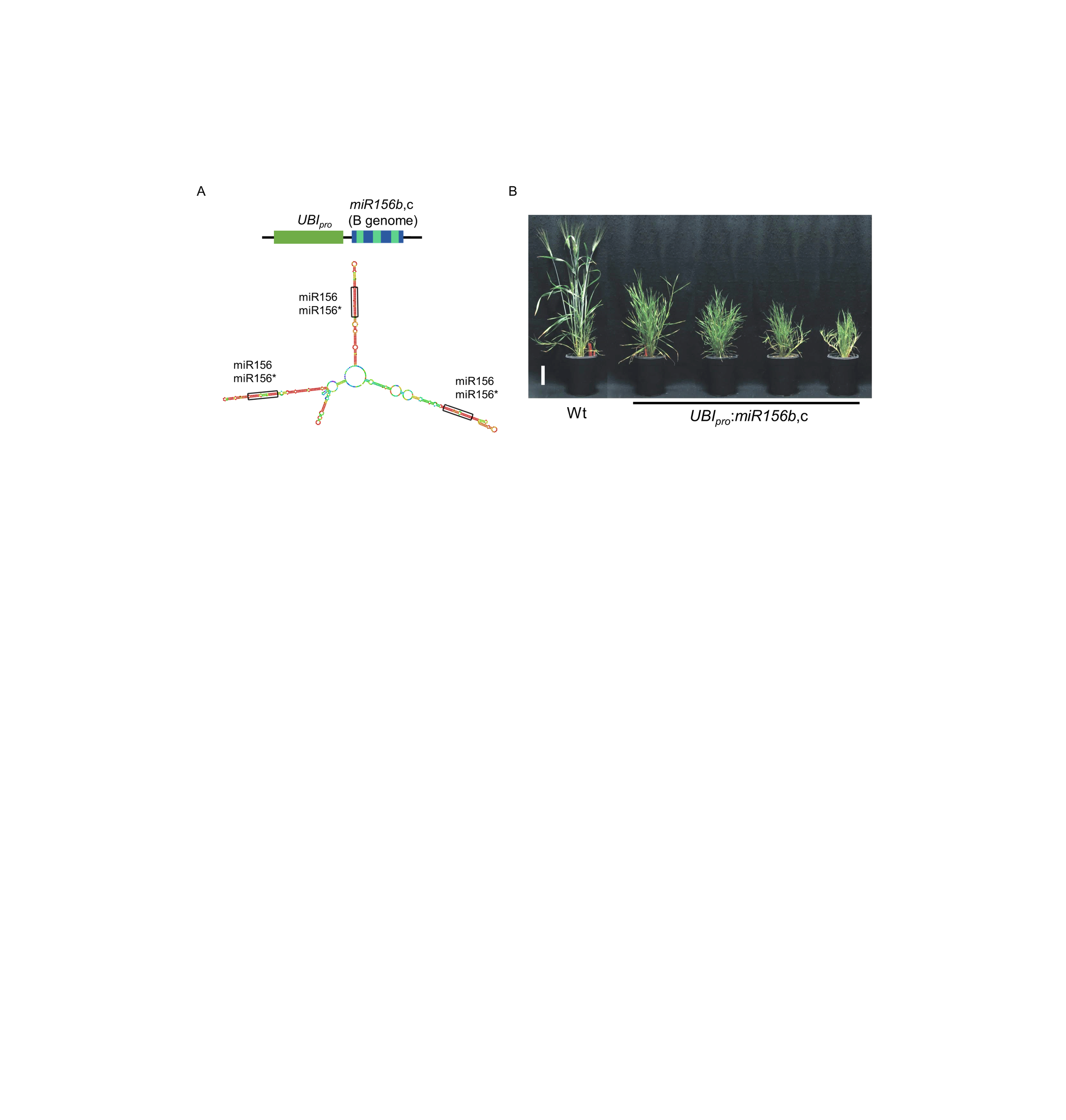

Supplement: S6 Fig — (A) Scheme showing the cassette including the maize UBIQUITIN promoter (UBIpro) and the sequences corresponding to the miR156b,c (B genome) locus. The predicted secondary structure for the miR156b,c sequence is shown below. Note the three stem-loop structures, including the three miR156/miR156* duplexes, corresponding to three miR156 precursors in tandem. (B) Wild type Kronos plant (Wt, left) and four independent transgenic T0 lines expressing UBIpro:miR156b,c grown under LD. Scale bar = 10 cm. (TIF) [file pgen.1010157.s009.tif]

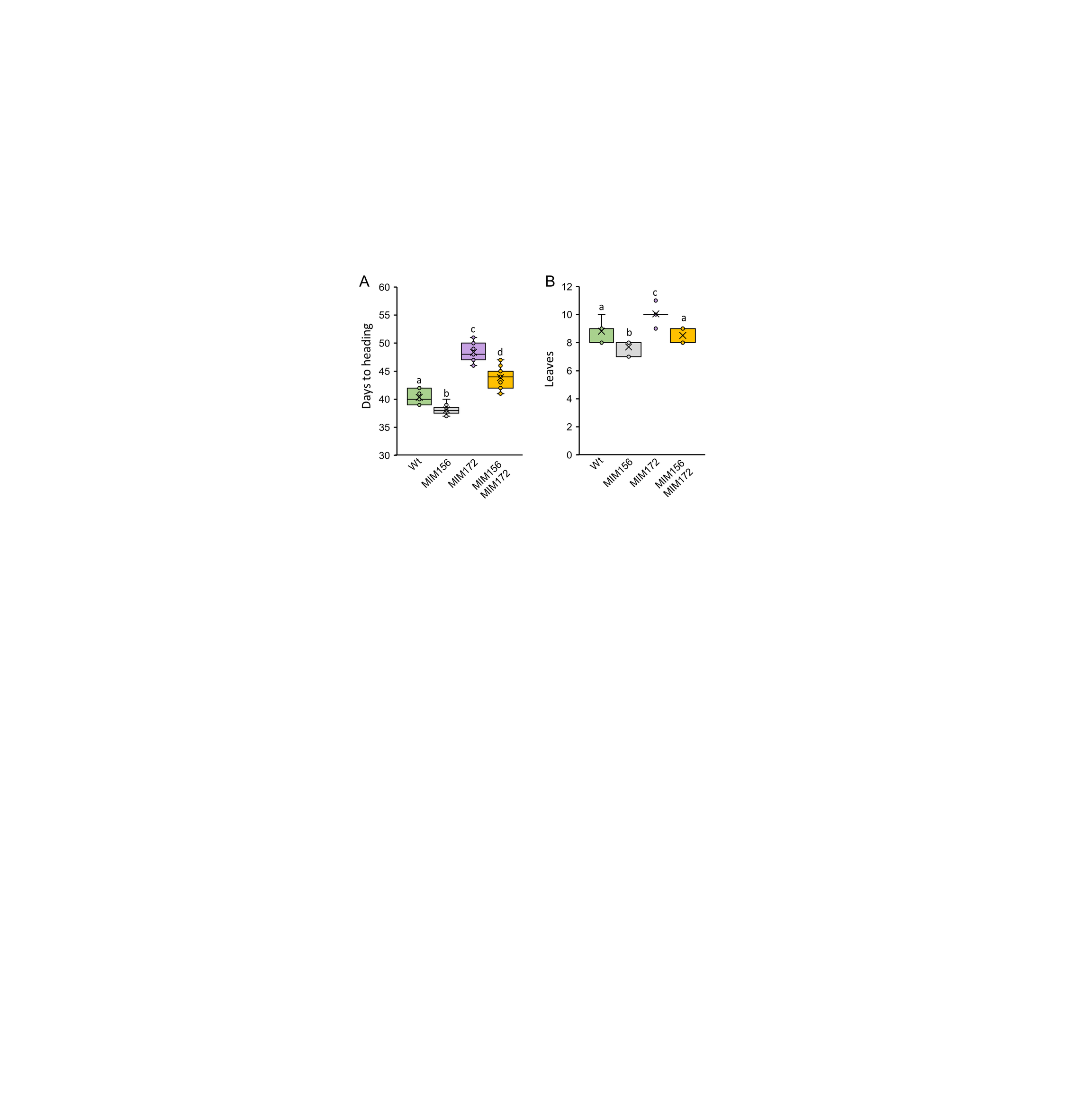

Supplement: S7 Fig — (A-B) Box plots showing days to heading (A; n ≥ 9) and the number of leaves produced by the main tiller before the transition to the spike (B; n ≥ 10) for wild type Kronos (Wt), MIM156, MIM172 and MIM156 MIM172 plants growing under LD conditions. Different letters above the box plots indicate significant differences (P ≤ 0.05) in pair-wise Kruskal-Wallis tests. Raw data and statistical tests in Data L in S1 Data. (TIF) [file pgen.1010157.s010.tif]

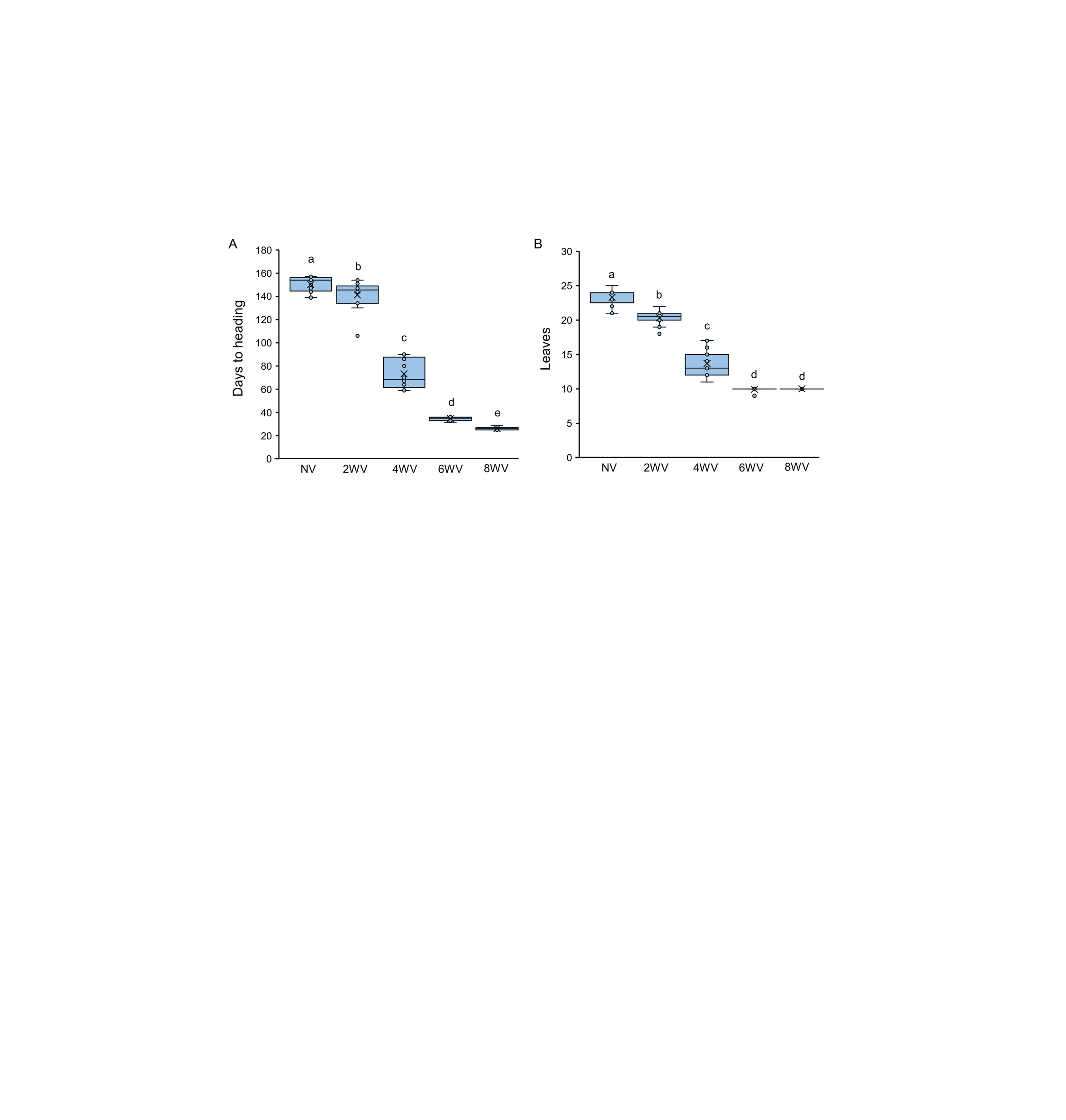

Supplement: S8 Fig — (A-B) Box plots showing the days to heading (A) and the number of leaves produced by the main tiller before the transition to the spike (B) for winter Kronos (vrn-A1) plants grown under LD conditions without vernalization (NV), with 2 (2WV), 4 (4WV), 6 (6WV) and 8 (8WV) weeks of vernalization. Different letters over the box plots indicate significant differences in Kruskal-Wallis non-parametric pairwise tests at P < 0.05. Raw data and statistical tests in Data M in S1 Data. (TIF) [file pgen.1010157.s011.tif]

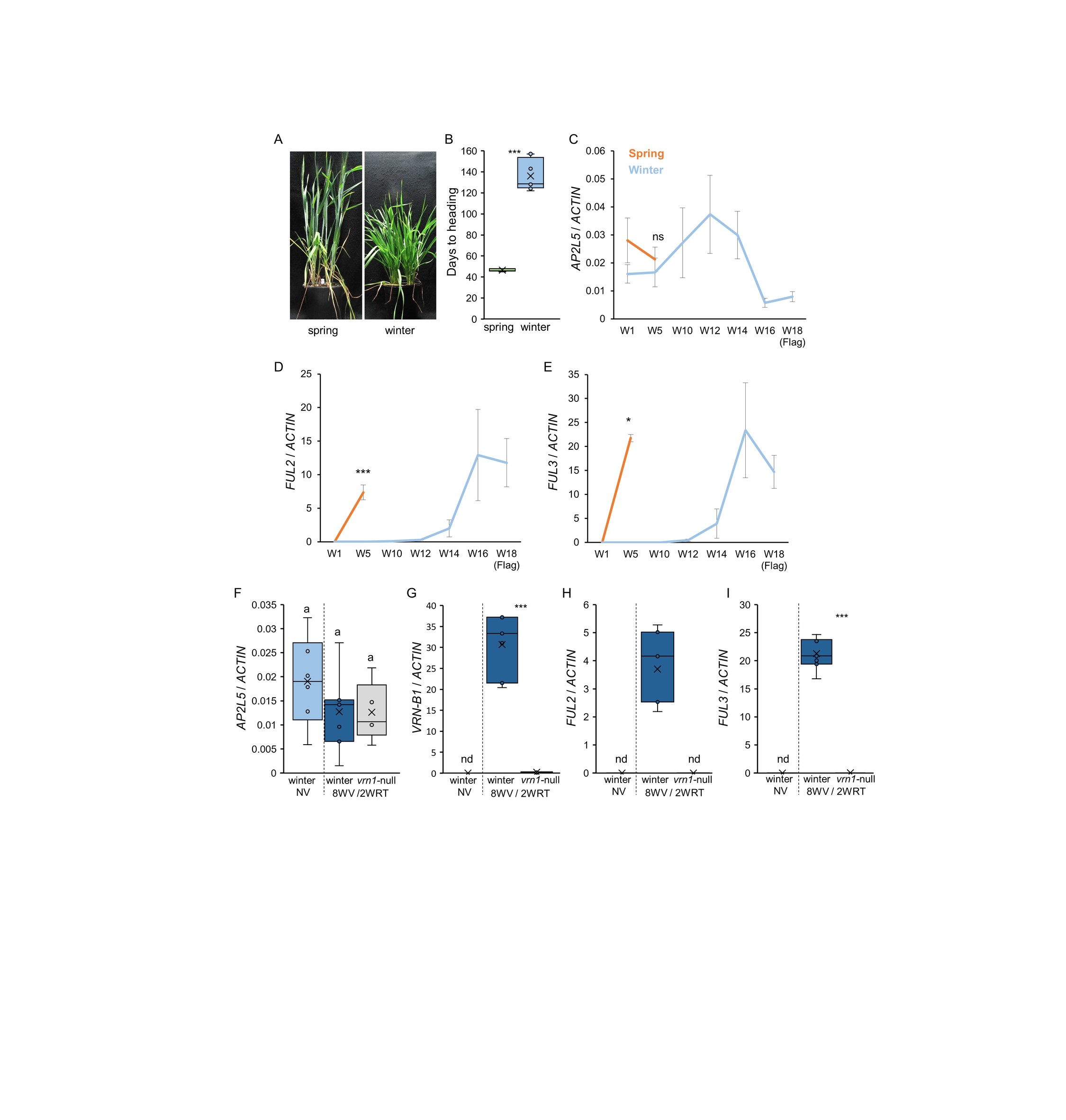

Supplement: S9 Fig — (A) Six-week-old spring and winter Kronos plants growing under LD conditions without vernalization. Scale bar = 10 cm. (B) Box plots showing the days to heading for spring and winter Kronos plants grown under LD conditions without vernalization (t-test, n ≥ 6). (C-E) Time course of AP2L5 (C), FUL2 (D) and FUL3 (E) expression levels determined by qRT-PCR in spring and winter Kronos plants grown under LD conditions in the absence of vernalization treatment. W1 = week1, 1st leaf; W5 = week 5, 7th leaf; W10 = week 10, leaves 10th-11th; W12 = week 12, leaves 13th-14th; W14 = week 14, leaves 15th-16th; W16 = week 16, leaves 18th-19th; W18, week 18, Flag leaves (20th-23rd). ACTIN was used as internal reference. Data correspond to four independent biological replicates. * = P ≤ 0.05 in t-test comparing expression at the same leaf in spring vs winter plants, except for panel (E) where a non-parametric Kruskal-Wallis test was used. (F-I) Box plots showing the expression of AP2L5 (F), VRN-B1 (G), FUL2 (H) and FUL3 (I) determined by qRT-PCR in the 7th (L7) leaf of winter Kronos without vernalization, and the 7th leaf of winter Kronos and vrn1-null mutants vernalized for 8 weeks and then moved to room temperature for two weeks. Samples from the 7th leaf were collected when the leaves were fully expanded. ACTIN was used as internal reference. Data correspond to at least 5 independent biological replicates. (F) Same letters indicate lack of significant differences in Tukey tests. (H) No expression of FUL2 was detected for the non-vernalized winter Kronos and the vrn1-null samples so no statistical test was performed. (G and I) No expression of VRN-B1 and FUL3 was detected for the non-vernalized winter Kronos sample, so a t-test was performed for the other two samples (*** = P < 0.001). Raw data and statistical tests in Data N in S1 Data. (TIF) [file pgen.1010157.s012.tif]

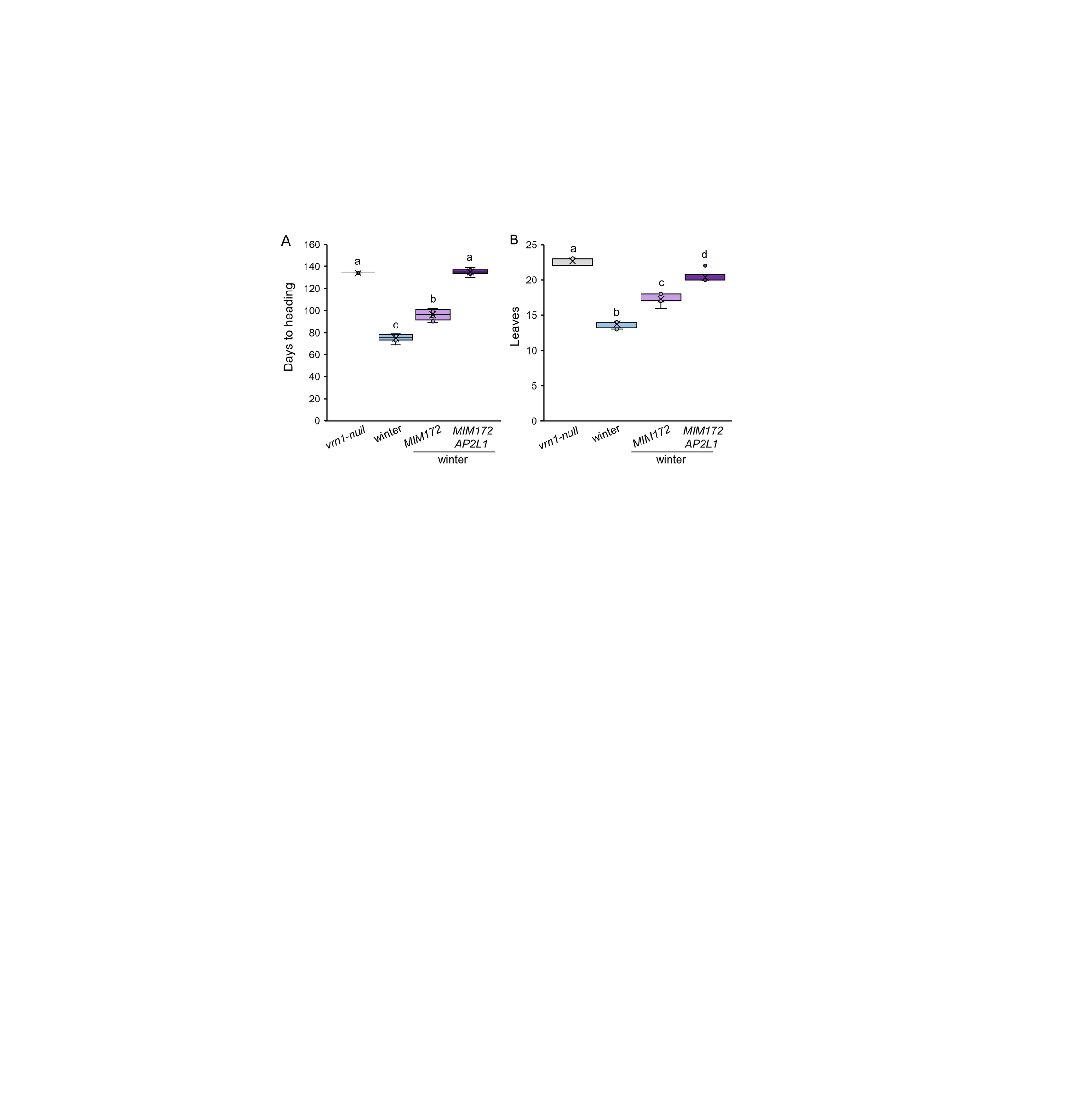

Supplement: S10 Fig — (A-B) Box plots showing days to heading (A) and number of leaves produced by the main tiller (B) in plants grown under LD conditions with 4 weeks of vernalization. vrn1-null = no functional VRN1 genes, winter Kronos, winter line with MIM172 transgene, winter line with MIM172 and UBIpro:AP2L-B1 transgenes. Different letters above the box plots indicate significant differences (P < 0.05) in Tukey tests. Raw data and statistical tests in Data O in S1 Data. (TIF) [file pgen.1010157.s013.tif]

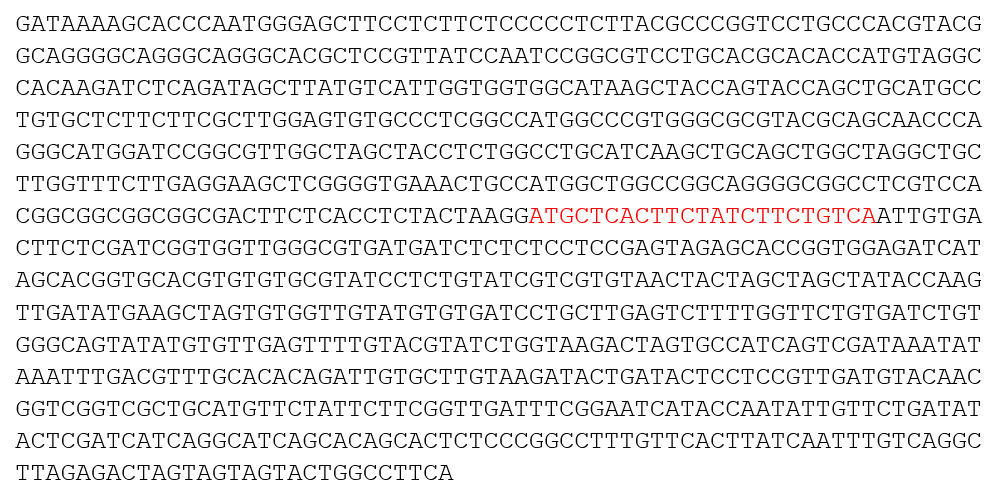

Supplement: S11 Fig — The sequence complementary to miR156 is in red. (TIF) [file pgen.1010157.s014.tif]
