# Supplementary material for: MiR172-APETALA2-like genes integrate vernalization and plant age to control flowering time in wheat
Source: PLoS Genet. 2022 Apr 25;18(4):e1010157. doi: 10.1371/journal.pgen.1010157 (PMC9037917; doi:10.1371/journal.pgen.1010157)
Supplement: Attachment — Submitted filename: Answers to reviewers and Editor comments.pdf [file pgen.1010157.s016.pdf]

Editor comments

Both reviewers are supportive of your work, but raise important considerations. Please deal with all their comments in a revision, especially the concerns of reviewer 2 about carefully describing which leaves you are using and the statistics. I would not suggest leaving out the mir156 data as suggested by reviewer 1.

Answer: We addressed both concerns in the answers to the specific reviewer's questions below.

Reviewer #1

This study examines the regulation of the flowering of wheat by two related APETALA2-like transcription factors, AP2L1 and AP2L5 (better known as Q). Two approaches were used to modify activity of AP2-like genes; (1) identification of knockout mutations directly in the genes and (2) altering the activity of a microRNA (miR172) that targets these genes for inactivation, through either transcript cleavage or post-translational mechanisms. The authors show that both of the AP2-like genes delay flowering of wheat. The authors also show that a second microRNA + transcription factor circuit likely acts upstream of the AP2-like genes (MiR156), as is the case in other plants. This is an interesting study of a worthwhile topic. It will be of interest to readers who are interested in the evolution of the networks that control plant development and those who are interested in crop improvement. The manuscript is clear and easy to follow. The authors present convincing genetic evidence to support the conclusion that AP2-like genes repress flowering of wheat.

Answer: Thanks for the positive comments.

A few points could be clarified or considered further before publication.

1. *Please consider the following point about the impact of AP2L genes on flowering and how this relates to vernalization: when do we conclude that a gene modifies vernalization requirement or vernalization response, versus influencing flowering time per se?*

*This question is relevant to the section starting at line **530** that suggests that miR172 modulates the vernalization response. Similarly, at line **826,** it is suggested that AP2L genes add an additional checkpoint to stop upregulation of FT1 prior to winter and at line **835** it is suggested that AP2L genes could be used to modify the vernalization requirement. An alternative view is that the AP2-like genes effect flowering time per se. That these genes do not impact the vernalization requirement or response and instead act in parallel by regulating FT1 irrespective of vernalization. And that the magnitude of the effect is dependent on context (genotype and growing conditions). The heading date data presented are consistent with this model. Irrespective of vernalization, over-expression of miR172 (or combined AP2L knockout mutations) accelerates flowering whereas MIM172 delays flowering.*
Put differently, would any gene that influences flowering time per se have a similar effect? Would any gene that regulates FT1 activity not elicit a similar pattern?

Answer: We agree with the reviewer that it is not easy to separate a direct effect on the vernalization response from an indirect effect through an impact on flowering time. Therefore, we have rephrased all the sections mentioned by reviewer one, that stated that miR172 and *AP2L* modulate the vernalization response. We have rephrased those sections to say that "miR172 and *AP2L* genes modulate the flowering response to different vernalization treatments". We have modified the abstract (L37) and several parts of the manuscript with the alternative wording indicated below.

L37. Abstract: "Interestingly, the levels of miR172 and both *AP2L* genes modulate the flowering response to different vernalization treatments in winter cultivars."

L538-539 (title): "miR172 modulates flowering time in winter wheat plants exposed to different vernalization treatments"

L570-571: "This result confirmed that miR172 modulates the flowering response under sub-saturating vernalization treatments in winter wheat".

L617-618 (title): "*AP2L1* and *AP2L5* play significant roles in the modulation of flowering time in winter wheat exposed to sub-saturating vernalization treatments."

L663-664: "... which in turn can modulate the flowering response to sub-saturating vernalization conditions."

L846-848:" However, in winter wheat plants, the upregulation of miR172 and the downregulation of *AP2L* genes requires the induction of *VRN1* during vernalization, which results in an additional regulatory layer that delays the upregulation of *FT1* and prevents premature flowering in the fall".

L856-857: "Our results also show that modulation of the *AP2L* dosage or activity may be a valuable tool to modulate the flowering response of winter cultivars as winters become milder due to climate change.".

The last sentence is supported by the significant interaction ($P < 0.0001$) observed between different vernalization treatments and genotypes for heading data and leaf number, even when the *vrn1*-null genotype was excluded (see new ANOVAs in Data F in S1 Data, supporting Fig 6). These results demonstrate that, depending on the level of saturation of the vernalization treatment, variation in miR172 is associated with different effects on heading time. Our wording was incorrect and we thank Reviewer 1 for helping us to be more precise in our language, but the interaction between miR172 levels and vernalization is well supported by the data.

We speculate this interaction is as follows: the *AP2L* genes are flowering repressors, and the repressing mechanism likely involves the repression of *FT1* expression in the leaves. In winter cultivars, the current vernalization model indicates that *VRN1* induction by cold promotes *FT1* expression in leaves by direct activation and indirectly by repressing *VRN2* expression. According to this model, and in combination with our own results, we suggest that changing *AP2L* levels would affect the threshold of *VRN1* expression required to promote *FT1* transcription and to trigger the *VRN1-VRN2-FT1* positive feedback loop. We know that *VRN1* expression increases the longer plants are in cold in a quantitative fashion. Therefore, in miR172 overexpression or in the *ap2l* mutants, the *VRN1* threshold required to induce *FT1* is likely lower, resulting in early flowering under non-saturating or non-vernalization conditions. By contrast, in plants with higher levels of *AP2L*, higher levels of *VRN1* would be required to induce *FT1* and flowering resulting in a longer vernalization requirement.

2. *Questions remain with respect to the miR156 section. Plant age might possibly regulate the timing of flowering of non-vernalized winter wheats, which flower only after extended periods. But most of the decrease in miR156 expression seems to happen in the first 5 weeks - are there any changes in winter wheat growth patterns after five weeks? (anything that can be described as a juvenile feature). Also, if the plant age pathway were critical, would you expect the AP2-like genes to be critical for the winter growth habit? Ultimately there was no functional test of whether miR156 can modify the flowering behavior of non-vernalized winter wheat. Maybe the miR156 section could be omitted.*

Answer: We were initially interested in testing the effect of the miR156 pathway on miR172 and *AP2L* genes in spring cultivars. In this background, where *VRN1* is induced in the absence of vernalization, our data showed that miR156 plays a critical role in regulating miR172-*AP2L* genes expression in leaves (Figures 3 and 4). In addition, we showed that the interactions between miR156 and miR172 play an important role in the regulation of wheat heading time (S7 Fig). We consider that this result is worth publishing.

Another result that we think is worth publishing is the decoupling of the induction of miR172 and downregulation of *AP2L* from the downregulation of miR156, and the role of *VRN1* in the downregulation of *AP2L*. As the induction of *VRN1* is a direct effect of vernalization, we speculate that *VRN1* induction acts as an additional regulatory layer between miR156 and miR172-*AP2L* to control the

flowering transition in winter cultivars. Non-vernalized winter plants with double mutations in *AP2L1* and *AP2L5* flowered 60 days earlier than wild type control, suggesting that controlling *AP2Ls* dosage or expression may be important for the winter growth habit.

Regarding the reviewer's comments about the role of miR156 in the regulation of flowering in winter cultivars, we have preliminary data showing an acceleration of flowering in non-vernalized MIM156 winter wheats. However, we still do not know how much of that acceleration depends on miR172-*AP2L*. Also, we do not know if the accelerated heading is associated with the initial downregulation of miR156 (from week 1 to week 5), or with the more gradual reduction observed after 5 weeks. Since this paper is focused on the miR172-*AP2L* module, we prefer to exclude this preliminary result until we can confirm it, and until we complete the crosses required to test this connection.

We also have preliminary information about the reviewer's question about the changes in winter wheat growth patterns. After 5 weeks, the growth pattern of spring and winter cultivars differed. The main tiller of spring plants continued generating leaves at the same rate until the spike emerge, whereas in winter cultivars growth of the main tiller slowed down and lateral tillers started to develop. We believe that this change in growing pattern is likely associated with the flowering transition of the SAM in spring plants, and the continued vegetative nature of the SAM in the winter wheats.

We also observed that leaf width increased in both spring and winter cultivars during the first 4 weeks of growth until L6, after which the width remained constant in the subsequent leaves. In the spring wheat lines transformed with MIM156, juvenile leaves were wider than controls, which agrees with the known role of miR156 controlling the juvenile-adult transition. However, we think these observations are not relevant to the story presented in this paper that is focused on the miR172-AP2L effect on heading time.

We agree with Reviewer 1 that many questions remain regarding the effects of miR156 (particularly in winter wheat), but this is not the focus of this paper. We think that the information provided about the interactions between miR156 and miR172 on heading time in spring wheat, the decoupling of this connection in winter wheat, and the identification of the role of *VRN1* in the downregulation of *AP2L* are worth publishing, and we prefer to follow the editor's suggestion to keep the miR156 results in the paper.

Reviewer #2

The manuscript describes the analysis of a large number of wheat mutants and transgenic lines to elucidate how the conserved microRNA miR172 and its targets APETALA2-like (AP2L) genes interact with the vernalization pathway involving VERNALIZATION 1 (VRN1), VRN2 and FLOWERING LOCUS T 1 (FT1 =VRN3) genes. The paper includes a lot of interesting data which is clearly described.
I only have a few detailed comments and suggestions.

1. *Methods: How long was the vernalization treatment?*

Answer: Vernalization treatments of different lengths were used in the different experiments and are now described in the legend of each of the figures:

- Fig 5A-F: no vernalization.
- Fig 5G-K: plants were vernalized for 8 weeks and then returned for 2 weeks at room temperature.
- Fig 6A and B: plants were vernalized for 2, 3 and 6 weeks.
- Fig 6C-H: plants were vernalized for 3 and 6 weeks.
- Fig 7: plants were vernalized for 2 weeks.
- S8 Fig: plants were vernalized for 2, 3, 6 and 8 weeks.
- S9A-E Fig: no vernalization.

- S9F-I Fig: plants were vernalized for 8 weeks and then returned for 2 weeks at room temperature.
- S10 Fig: plants were vernalized for 4 weeks.

2. *The authors present a lot of expression data in different figures of the manuscript. In many diagrams, but not in all the authors show expression levels of target genes in different leaves. Please, describe in the Methods section how and when the leaves were harvested, i.e. the 3rd, 5th, 7th leaf were harvested whenever they were fully developed or they were harvested at one time point (when and what time of the day). If the data is available it would be informative to add information on what stage the plants were in when leaves were harvested.*

Answer: We added in the Methods section: "In all experiments we collected the last fully expanded leaf in the main tiller, and harvested samples 4 hours after lights were turned on, except in Figure 6 where leaves were harvested in the middle of the photoperiod (ZT8)."

3. *In some figures, there is no information on which leaf/or leaves were used for the expression analysis. For example, Fig. 1H the legend says: Box plots showing the expression levels of AP2L1 in leaves (H; n = 4).*

Answer: Thank you for pointing out this omission. The Fig1H and M legends were corrected as follow: "(**H-I**) Box plots showing the expression levels of *AP2L1* in the 5th leaves (**H**; n = 4)" and "… and expression levels of *AP2L1* in the 5th leaves (**M**)"

4. *Fig. 1H, I, J , Fig. 2 etc. Box plots comparing more than two genotypes/conditions: please provide information on sig. differences with a, b, c. etc to distinguish between all different factors (genotypes) rather than only showing comparisons to the WT (asterisks) as it was done for example in Figure 3.*

Answer: We converted all mean comparisons in the main text, figures and supplementary figures to Tukey tests for consistency, and to facilitate comparisons among any means. However, since the Dunnett tests are more sensitive and provide additional information on the level of significance of the differences against the WT (rather than a fixed *P* value), we kept the Dunnett and Tukey results in the Supplemental Data for readers who might be interested in this additional information. In the text and figures we only refer to the Tukey tests presented in the figures.

5. *Figure 4: the authors show differences in expression between WT and MIM156 lines for VRN1 and FT1 in leaves 3, 5 and 7. Significant differences were only observed in the 3rd leaf. What was the developmental stage of the shoot apex of the two genotypes at the 3-leaf stage and what were the differences between the genotypes? As the manuscript contains a lot of data, I was wondering if it would be sufficient to show the expression data only for leaf 3 (unless you want to stress here also the expression changes over development for the different genes).*

Answer: In Figure 4E-I, we show expression data from L1, L3 and L7. We included those three time points because we are interested in showing the dynamic changes in the expression profiles of these genes during plant development. We also think that it is important to provide L1 as a baseline, and L7 to show the marginally significant differences for miR172 (*P* = 0.05) and the significant differences for *AP2L1*. Although not significant, the L7 data also shows a slight increase in *VRN1* and *FT1* expression that is consistent with the decrease in *AP2L1*. We have not checked the SAM in these plants, but based on our own experience and previously published experiments (Shaw et al. PLoS Genetics 2020, 16: e1008812, Supplemental Figure S1), we know that in Wt Kronos the SAM transitions to the double ridge stage at leaf 4 (L4), so the plants at L7 are certainly at the reproductive stage.

6. *Fig. 5A-F: Here expression levels in winter and spring Kronos in different weeks are displayed. The legend says: W1= week1, 1st leaf; W5= week 5, 7th, leaf; W10= week 10, leaves 10th-11th; etc. I was wondering if in week 5 this was the 5th leaf for the winter Kronos and the 7th leaf for the spring Kronos?*

Answer: In our growth chamber conditions, during the first 4 weeks, spring and winter cultivar display similar phenotypes and growing patterns. Therefore, we were able to collect the fully expanded leaf 7th during the 5th week in both genotypes. However, it is important to mention that leaf samples were not collected on the same day (34 days for spring and 36-38 days for winter plants). We did notice that after this point, the rate at which leaves emerged on the main tiller of winter plants slowed down compared to spring plants, which continue to produce leaves at the same rate until the spike emerge at 6-7 weeks. We think that this change in plastochron likely reflects the different developmental trajectories of the SAM in both genotypes.

We added a note to the Fig 5 legend: "In both genotypes the fully expanded 7th leaves were collected during the 5th week. However, leaf samples were not collected on the same day (34 days for spring and 36-38 days after planting for winter)."

7. *In other words, I assume that in W5 the two genotypes were at very different developmental stages. In this context, I was wondering if the sig. tests- comparison of expression levels between genotypes at each time point (W1, W5) makes sense. I think in these figures it would be more important to show sig. difference in expression levels between weeks within each genotype. Also to support the following interpretation.*

Answer: We added a statistical comparison in the Supplemental data between spring W5 and winter W18, and as the reviewer suggested, the differences were not significant for any of the six genes in Figures 5A-F and two of the genes in S9D-E Fig. The differences were only marginally significant for *AP2L5* ($P = 0.03$). We added in the text: "No significant differences in gene expression were detected between the spring line at W5 and the winter line at W18 in Figs 5A-F (Data E in S1 Data) and S9D-E (Data N in S1 Data), suggesting a similar regulation of these genes at heading time for the two genotypes".

8. *The authors write: In winter lines, we observed a progressive increase in the expression of miR172 with age until week 16 (Fig 5E). Interestingly, we did not observe a parallel downregulation of the transcript levels of AP2L1 (Fig 5F) and AP2L5 (S9C Fig) after W5. This result is similar to the limited effect of UBIpro:miR172 on AP2Ls transcript levels described in S2A and B Fig. After W14, we observed an abrupt downregulation of AP2L1 and AP2L5 that coincided with the up-regulation of VRN1 (Fig 5C), and FUL2 and FUL3 (S9D and E Fig). Please, add here in the text that it also coincides with the strong upregulation of FT1.*

Answer: We added *FT1* to the text: "After W14, we observed an abrupt downregulation of *AP2L1* and *AP2L5* that coincided with the up-regulation of *FT1* (Fig 5B), and the *SQUAMOSA* genes …".

9. *Further, please, provide statistical evidence for the significant down/up regulation of VRN1 and AP2 in week 18.*

Answer: We added statistical comparisons for the differences in time in Figure 5A-F and S9C-E Fig (Data E in S1 Data). The differences in time were significant for all genes except *AP2L5*. For *FT1* and *VRN1* we did not detect expression until W14, so we included only the last three time points in the ANOVAs. Similarly, for *FUL2* no expression was detected until W10 and for *FUL3* until W12.

We performed the requested Tukey tests suggested by the Reviewer 2, which confirmed that both the upregulation of *VRN1* and downregulation of *AP2L1* at W18 were significant (see Tukey graphs of transformed data below). However, comparisons among specific time points are usually not provided in time courses because samples are not independent, so we prefer to exclude these mean comparisons from the paper. We think that the addition of the significant ANOVAs presented in the Supplemental Data together with the trends showed in the figures are sufficient to demonstrate that all these genes are developmentally regulated. At the end of the description of the results of Figure 5A-F and S9D-E we added:

"For all the genes in Figs 5A-F (Data E in S1 Data) and S9D-E (Data N in S1 Data), the ANOVAS for the differences in gene expression along development were significant for both spring and winter lines, confirming that all these genes are developmentally regulated. Only *AP2L5* (S9C Fig) showed no significant differences in time for the spring line and only marginally significant differences for the winter lines ($P = 0.05$).

Tukey tests among different time points showing expression for *VRN-B1* and *AP2L1*. Not included in the paper.

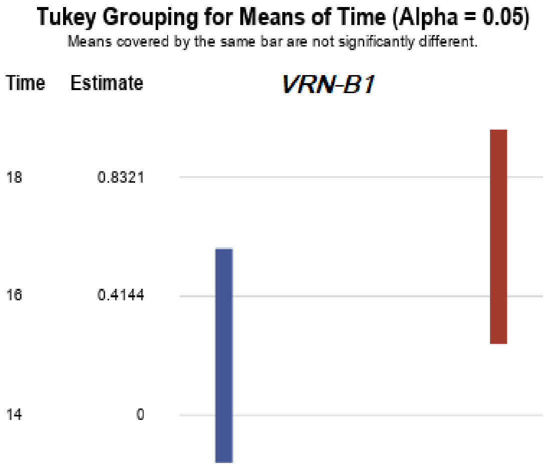

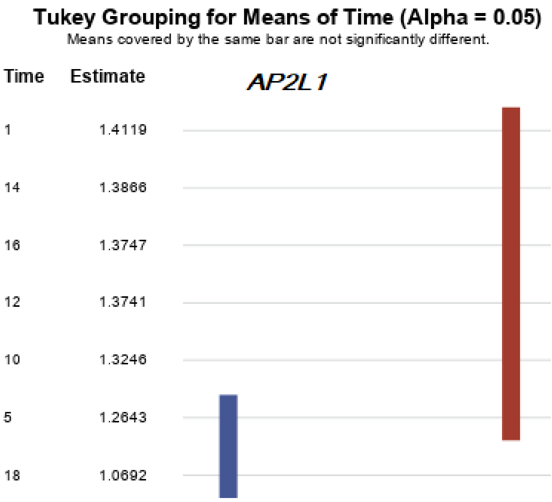

***10.** Here again it would be good to provide information on the developmental stage of the spike at W18 (and W16). What do you think could be the role of the AP2 downregulation in W18 for spike development (as far as I can see there are no differences in AP2 expression from W1-W16).*

Answer: Unfortunately, we did not dissect plants to check the status of the SAM at W18. However, since at this time point we collected samples from flag leaves, it is safe to infer that the SAM already transitioned to reproductive development.

Regarding the possible role of the downregulation of *AP2L* genes at W18, we think it might be related to the later roles of these genes. Initially, *AP2L* genes delay the time of the SAM transition to the reproductive phase (supported by the lower number of leaves of *UBIpro:miR172* plants and *ap2ls* mutants). Later, miR172 and *AP2L* genes also control spike and spikelet development [66, 84] and stem elongation. We have previously observed that MIM172 plants have shorter internodes, while *UBIpro:miR172* are taller [66]. Similar observations were done recently in barley, where the dominant miR172-insensitive *Zeocriton* mutant (*AP2L2*) negatively regulates internode and peduncle elongation [95]. Based on these results, we speculate that the downregulation of *AP2L* may play a role in internode elongation. We have previously shown that *FT1* promotes GA biosynthesis and prevents GA degradation in the developing spikes [92]. Therefore, the downregulation of *AP2L*s at later stages may facilitate the upregulation of *FT1* in the leaves, which will impact its role on stem elongation. We have added the following sentence to the Discussion: "We speculate that the downregulation of *AP2Ls* at later developmental stages could reflect an additional role of these genes on internode elongation. Transgenic wheat plants expressing MIM172 have higher *AP2Ls* expression and shorter internodes than the non-transgenic controls [66]. Similarly, a dominant mutation in the miR172 target site of *AP2L2* in barley negatively regulates peduncle elongation during the reproductive transition [95]. Since the downregulation of *AP2Ls* in flag leaves was associated with an increase in *FT1* expression (Fig 5), and *FT1* is required to promote internode elongation in wheat [92], we speculate that *AP2L*s may control internode elongation by controlling *FT1* expression in the leaves.."

11. *Please, also consider if the expression plots would look much more alike between spring and winter Kronos if you would display W1 and W5 in spring and W1 and W18 in winter as W5 in spring and W18 in winter Kronos may actually be comparable stages.*

Answer: We added the statistical comparison between W5 in spring wheat and W18 in winter wheat in the main text and in Data E and N in S1 Data, and described the result in our previous response to question 7. We think that it would be misleading to eliminate the intermediate time points in winter wheat. In the current format the readers can compare the final time points in spring and winter, and the statistical comparison is described and the data provided. If we present only the final time points some information will be lost, so we prefer to present the graph as it is.

12. *The figures 5 G-K nicely show that VRN1 indeed regulates AP2 and following figures that AP2 and miR172 modify the vernalization response. Do you think that AP2 and miR172 modulate VRN1 expression independent of FT1 as suggested in your model? Or would it make sense to include FT1 in your Fig. 8A and B?*

Answer: There is evidence in Arabidopsis that AP2Ls directly regulate *FT* expression [56,57]. In wheat, it has been shown that *FT1* can bind to the *VRN1* promoter [17,18] and in *Brachypodium* that downregulation of *FT1* results in the downregulation of *VRN1*. Moreover, in this study we show that miR172 can regulate *FT1* expression independently of *VRN1* and *VRN2*. Combining all these results, we think that the most parsimonious hypothesis is that *AP2L* genes regulate *VRN1* through its effect on *FT1*, as suggested in the model presented in Fig 8A. However, we cannot rule out a more direct connection or effects mediated by other factors.

FT1 is included in Figure 8A, but is not presented in Fig 8B because it is redundant with *VRN1* expression and because the objective of Fig 8B is to show the expression profiles of miR172 and *AP2L1* and their relation with *VRN1* in spring and winter wheat (vernalized and not vernalized). It is known that vernalization operates directly on *VRN1* and only indirectly on *FT1*.

13. *Some of the links in the model (PhyC, PPD1 etc.) are not derived from data in this paper. You may want to cite the appropriate literature in Fig. 8.*

Answer: Excellent suggestion! We have modified the figure to include the appropriate references supporting each of the arrows in the model. We indicated the Arabidopsis references in red so the reader can rapidly identify which of the arrows are supported by information in wheat or other grasses versus a distantly related dicot species.
